# Supplementary material for: Myths in psychology: psychological misconceptions among Spanish psychology students
Source: PeerJ. 2022 Aug 11;10:e13811. doi: 10.7717/peerj.13811 (PMC9375966; doi:10.7717/peerj.13811)

SUPPLEMENTARY MATERIAL

Table 1.

Sample distribution of participants according to academic year (1-4) and University (face-to-face, UAM and online, UNED).

University / Year	Face-to-face	Online	TOTAL
1st	139 (38%)	325 (59%)	464 (51%)
2nd	109 (30%)	111 (20%)	220 (24%)
3rd	59 (16%)	52 (9%)	111 (12%)
4th	57 (16%)	64 (12%)	121 (13%)
TOTAL	364	552	916

Table 2

Items used in the questionnaire and wheter were used by Gardner and Brown (2013). All items, used by Gardner and Brown (2013) and the new items that we included, were extracted from the book 50 Great Myths of Popular Psychology (Lilienfeld et al., 2010).

**Item**	**Presented in Gardner & Brown, 2013**
1. Most people use only about 10% of their brain power.	Yes
2. Almost all color-blind people can see at least some colors. (*)	Yes
3. Some people are exclusively left-brained while others are right-brained.	Yes
4. Extrasensory perception is NOT a real phenomenon. (*)	No
5. Subliminal messages can't persuade people to perform some behaviors. (*)	No
6. Humans have an invisible body energy that can cause psychological problems when blocked.	No
7. People become increasingly satisfied with their lives in old age. (*)	Yes
8. Most adopted children are psychologically healthy.	Yes
9. Married couples enjoy more marital satisfaction after they have children.	Yes
10. Infants establish attachment bonds only to their mothers.	Yes
11. Playing classic music to infants DON'T boosts their intelligence. (*)	No
12. When dying, people DON'T pass through a universal series of psychological stages. (*)	No
13. Individuals commonly DON'T repress the memories of traumatic experiences. (*)	Yes
14. People with amnesia can still recall some details of their earlier lives. (*)	Yes
15. The memory of everything we've experienced is stored permanently in our brains, even if we can't access all of it.	Yes
16. With effort, we can remember events back to the time of our birth.	Yes
17. Some people have true photographic memories.	Yes
18. Human memory works like a tape recorder, and accurate records events we've experienced.	No

19. Rote memorization is NOT the best way to retain information. (*)	No
20. IQ scores are relatively unstable during childhood. (*)	Yes
21. There is a modest correlation between brain size and IQ in humans. (*)	Yes
22. As a general rule. students typically recall only 10% of what they read.	Yes
23. Irregularly provided feedback best promotes long-term learning. (*)	Yes
24. Negative reinforcement is a type of punishment.	Yes
25. Students learn best when teaching styles are matched to their learning styles.	No
26. Direct instruction is superior to discovery learning. (*)	No
27. Hearing material while we are asleep (sleep learning) can be an effective aid to learning.	Yes
28. Hypnotized people are aware of their surroundings and can recall the details of conversations overheard during hypnosis. (*)	Yes
29. It is impossible to lie under hypnosis.	Yes
30. Virtually all people dream. (*)	Yes
31. Our brains rest during sleep.	Yes
32. Researchers have demonstrated that dreams possess symbolic meaning.	No
33. Awakening a sleepwalker is NOT dangerous. (*)	No
34. The polygraph (lie detector) test is NOT an accurate means of detecting dishonesty. (*)	Yes
35. Ulcers are caused primarily by stress.	Yes
36. Women are NO better than men at accurately guessing the feelings of others. (*)	Yes
37. Unfamiliarity breeds contempt: We dislike things we have less exposure to. (*)	Yes
38. Positive thinking is better than negative thinking for all people.	No
39. Voice stress analyzers can help to detect lying.	No
40. We are most romantically attracted to people who are similar to us. (*)	Yes
41. The more people present at an emergency, the greater the chance that someone will intervene.	Yes

42. Expressing anger directly toward another person or object makes us more aggressive. (*)	Yes
43. Groups tend to make less extreme decisions than individuals.	Yes
44. The best way to change someone's attitude is to give them a large reward to do so.	Yes
45. Men and women communicate in completely different ways.	No
46. People's attitudes are NOT highly predictive of their behaviors. (*)	No
47. We CANNOT tell a person's personality by merely looking at their handwriting. (*)	Yes
48. Knowing a person's astrological sign predicts their personality traits at better than chance levels.	Yes
49. Most people who were physically abused as children DON'T go on to become abusers themselves. (*)	Yes
50. Most children survive the divorce of their parents without much, if any, long-term psychological damage. (*)	Yes
51. Obese people are more cheerful ("jolly") than thin people.	Yes
52. The fact that a trait is heritable means we CAN'T change it.	No
53. People's responses to inkblots tell us a great deal about their personalities.	No
54. Only deeply depressed people commit suicide.	Yes
55. People with schizophrenia DON'T have multiple personalities. (*)	Yes
56. There has recently been a massive epidemic of childhood autism.	Yes
57. All clinically depressed people suffer from extreme sadness.	Yes
58. Most people who experience severe trauma, (e.g., as in military combat) DON'T develop posttraumatic stress disorder (PTSD). (*)	Yes
59. Psychiatric hospital admissions and crimes DON'T increase during full moons. (*)	No
60. Hallucinations are almost always a sign of serious mental illness.	No
61. Homicide is more common than suicide.	Yes
62. Most rapes are committed by strangers.	Yes
63. The words "insanity" and "sanity" are purely legal NOT psychological terms. (*)	Yes

64. Most people that plead insanity are NOT faking mental illness. (*)	Yes
65. Most psychopaths are violent.	No
66. Rehabilitation programs have NO effect on the recidivism rates of criminals.	No
67. Crowding consistently leads to more aggression.	No
68. More experienced therapists are generally NO more effective than those with little experience. (*)	Yes
69. Most psychotherapy involves a couch and exploring one's early past.	Yes
70. Antidepressants are much more effective than psychotherapy for treating depression.	Yes
71. Taking a placebo (i.e. sugar pill) can change brain functioning and its chemistry. (*)	Yes
72. Electroconvulsive therapy is rarely administered today.	Yes
73. Expert judgment and intuition are the best means of making clinical decisions.	No
74. Most modern therapies are NOT based on the teachings of Freud. (*)	No
**Item used by Gardner and Brown (2013) and excluded from the questionnaire**	
Visual perceptions are accompanied by tiny emissionsfrom the eyes.	
Adult humans can grow new brain cells. (*)	
A small percentage of the elderly live in nursinghomes. (*)	
Extreme fear can turn our hair white	

Table 3

Relation of means and standard deviations (in parentheses) of the total score, according to the academic year.

	Total score on the scale				
	1st year	2nd year	3rd year	4th year	TOTAL
Face-to-face	178.60 (16.77)	179.89 (19.39)	172.63 (18.67)	173.86 (18.09)	177.27 (18.25)
Online	212.24 (20.55)	203.17 (19.01)	198.85 (24.27)	188.41 (21.07)	206.39 (22.15)
TOTAL	202.16 (24.84)	191.63 (22.43)	184.91 (25.09)	181.55 (20.95)	

Table 4

Summary of the Two-Factor ANOVA (university and academic year).

Source	Df	F	P	η_G^2
University	1	251.321	< 0.001	0.216
Year	3	19.295	< 0.001	0.059
University*year	3	8.35	< 0.001	0.027
Residue	908			

Table 5

Scientific dissemination sources indicated by the participants.

Scientific dissemination sources	Frequency
1. Databases for academic work	77 (33.47%)
2. Textbooks	27 (11.74%)
3. "Pop" Psychology Journals (e.g., Psychology and Mind)	72 (31.3%)
4. General media (scientific sections ABC, El País ..)	13 (5.65%)
5. Social media (Twitter, YouTube…)	59 (25.65%)
6. Other/undefined	9 (3.91%)

Table 6

Summary of the descriptive statistics of the total score based on the familiarity with scientific dissemination, both according to universities and in the total sample.

Scientific dissemination	Face-to-face			Online			TOTAL		
	N	Mean	SD	N	Mean	SD	N	Mean	SD
No familiarity	278	178.43	17.51	402	208.82	22.13	680	196.39	25.25
Familiarity	86	173.55	20.12	150	199.89	20.95	236	190.29	24.21

Figure 1. Relative frequencies of items 1-20

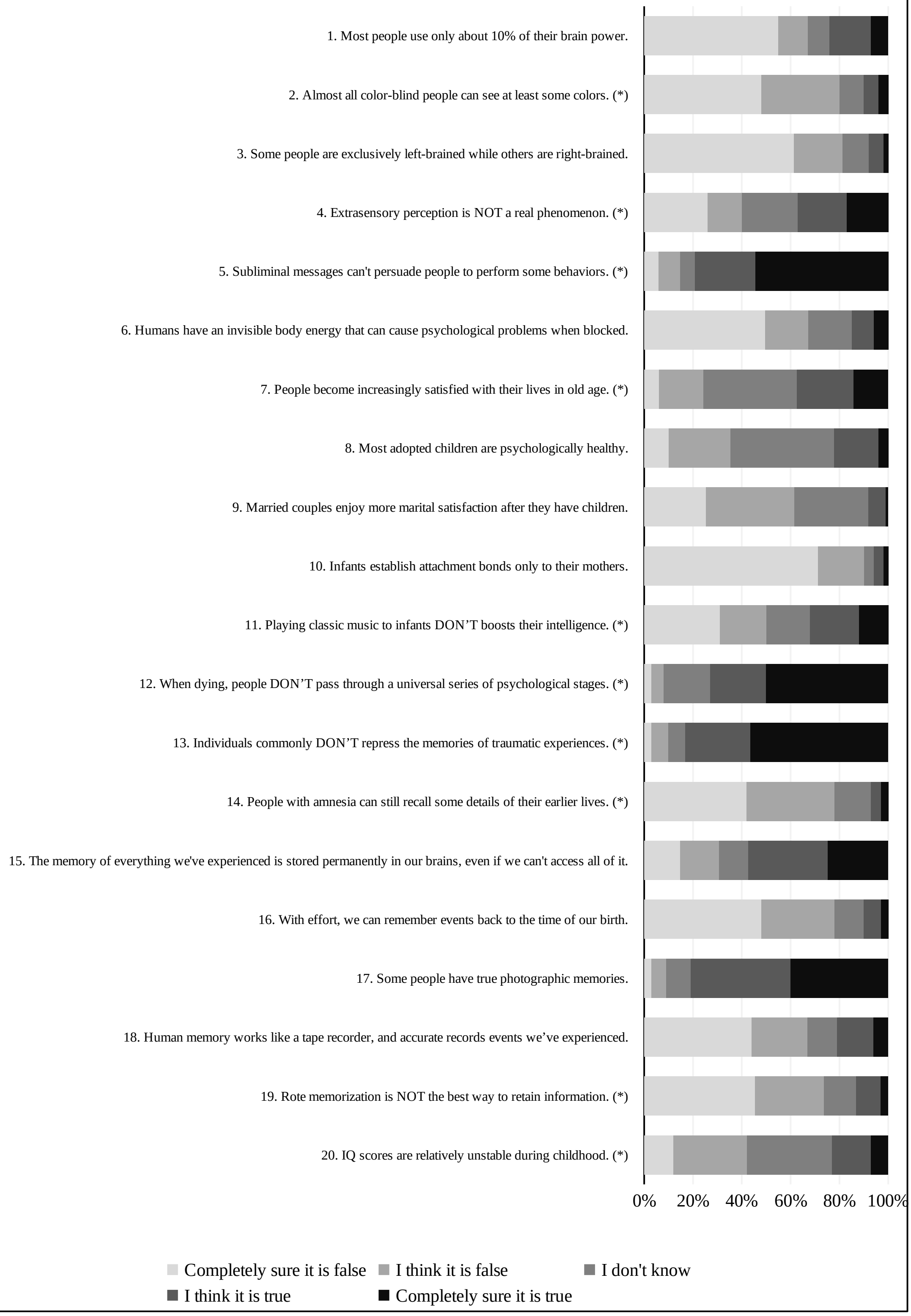

Figure 2. Relative frequencies of items 21-39.

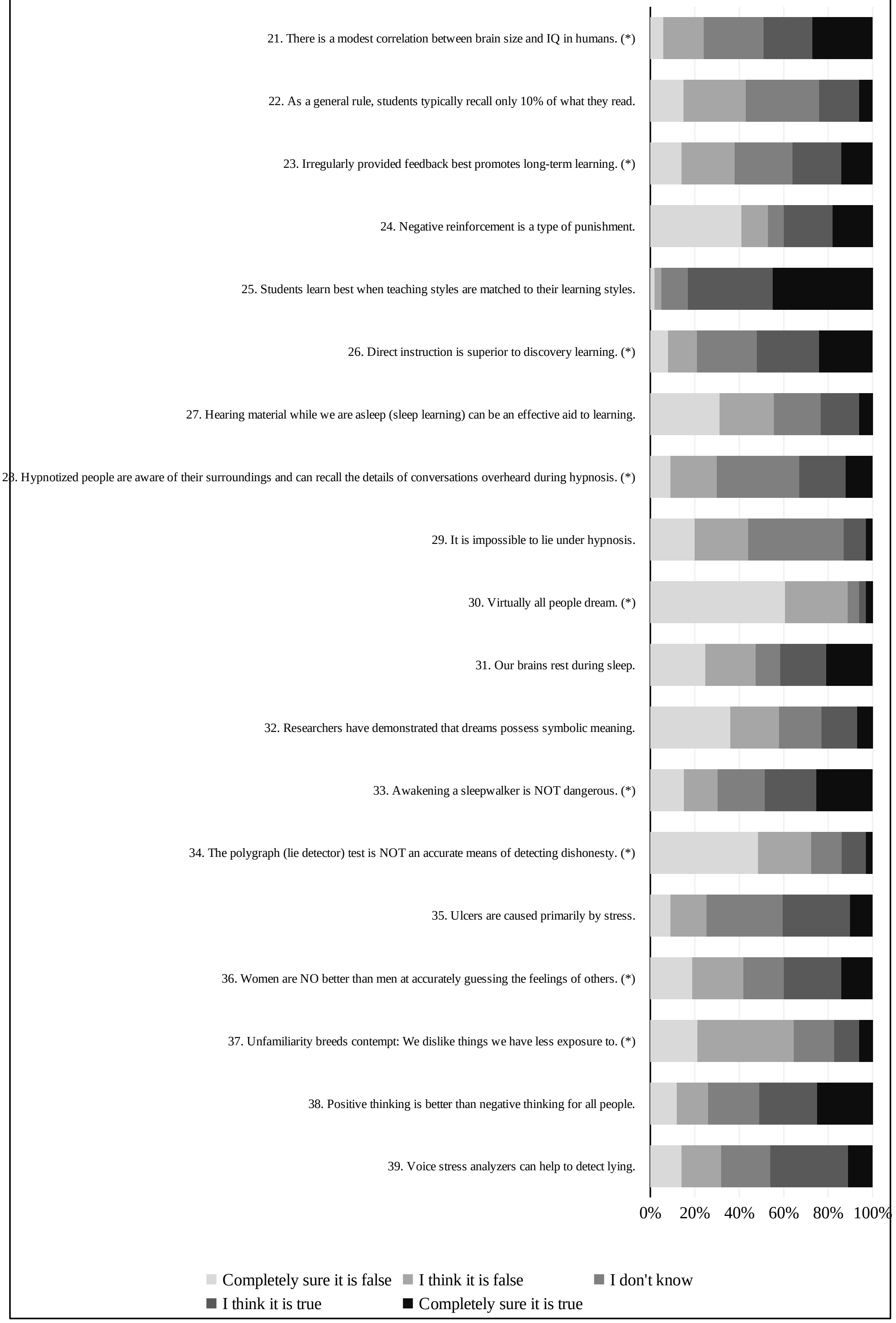

Figure 3. Relative frequencies of items 40-59.

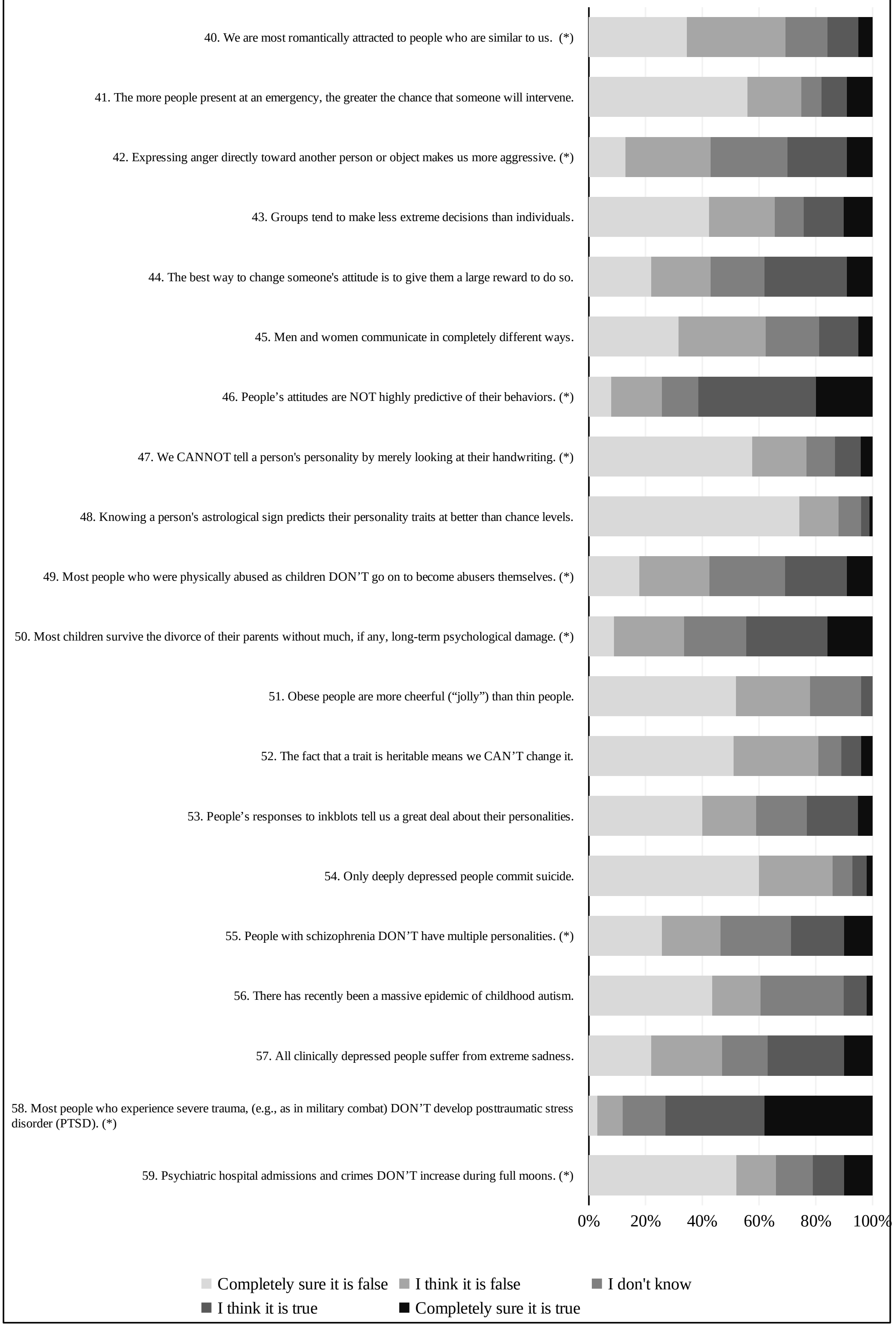

Figure 4. Relative frequencies of items 60-74.

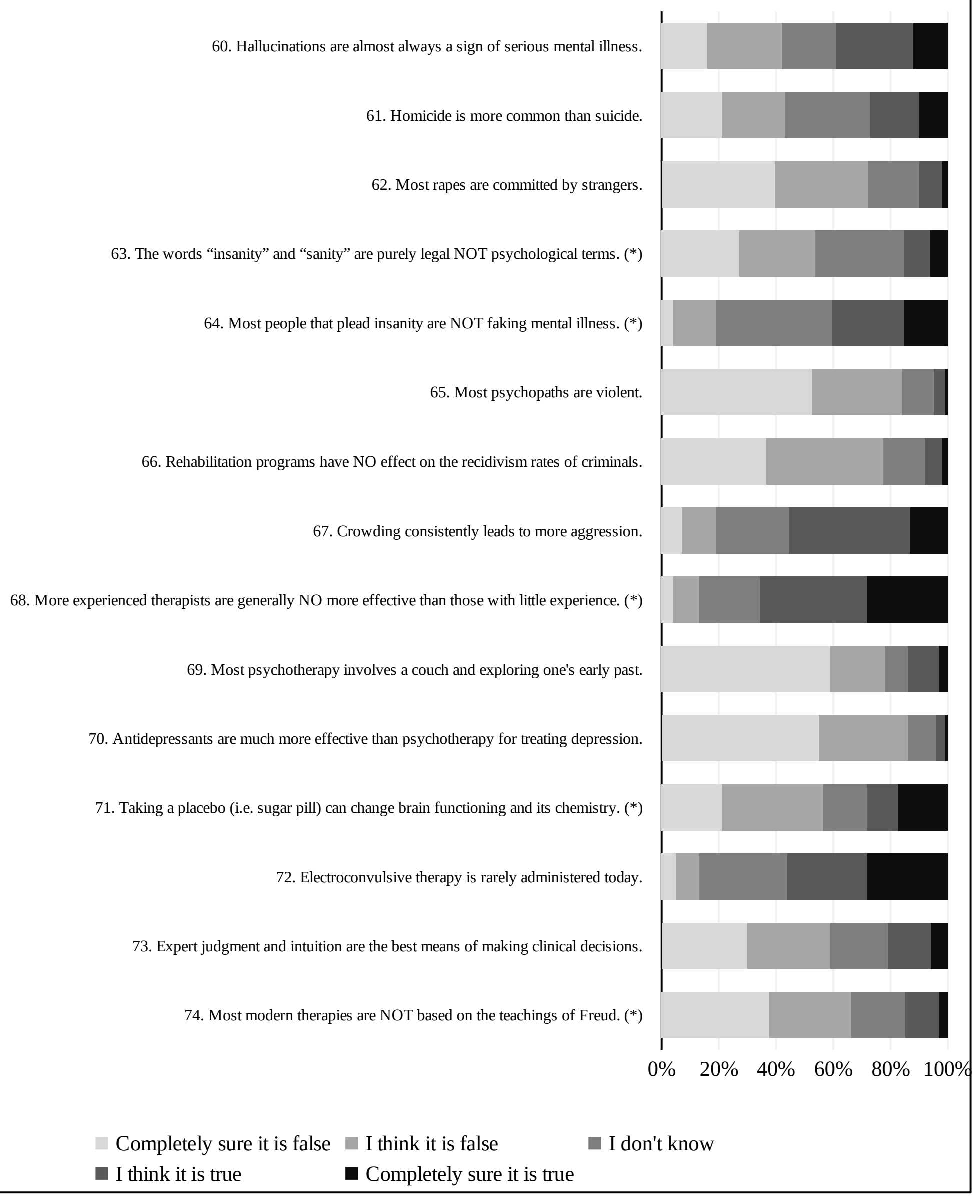

Supplement: Supplemental Information 2 [file peerj-10-13811-s002.pdf]
